# Local Infiltration Analgesia for Postoperative Pain Control following Total Hip Arthroplasty: A Systematic Review

**DOI:** 10.1155/2012/709531

**Published:** 2012-07-05

**Authors:** Denise McCarthy, Gabriella Iohom

**Affiliations:** Department of Anaesthesia and Intensive Care Medicine, Cork University Hospital, Cork, Ireland

## Abstract

Local infiltration analgesia (LIA) is an analgesic technique that has gained popularity since it was first brought to widespread attention by Kerr and Kohan in 2008. The technique involves the infiltration of a large volume dilute solution of a long-acting local anesthetic agent, often with adjuvants (e.g., epinephrine, ketorolac, an opioid), throughout the wound at the time of surgery. The analgesic effect duration can then be prolonged by the placement of a catheter to the surgical site for postoperative administration of further local anesthetic. The technique has been adopted for use for postoperative analgesia following a range of surgical procedures (orthopedic, general, gynecological, and breast surgeries). The primary objective of this paper was to determine, based on the current evidence, if LIA is superior when compared to no intervention, placebo, and alternative analgesic methods in patients following total hip arthroplasty, in terms of certain outcome measures. The outcomes considered were postoperative analgesia scores, joint function/rehabilitation, and length of hospital stay. Secondary objectives were to review available evidence and current knowledge regarding the pharmacokinetics of local anesthetic and adjuvant drugs when administered in this way and the occurrence of adverse events.

## 1. Methods

### 1.1. Literature Search

The National Library of Medicine's Medline database and the Cochrane Central Register of Controlled Trials were searched for the time period January 1, 1966 to present (last search performed 21st January 2012). The search terms used were local infiltration analgesia, joint infiltration, joint infusion, wound infiltration, continuous wound infusion and local anesthetic infusion, local anesthetic infiltration. These search terms were then combined with the term total hip arthroplasty/replacement and searched again. Searches were restricted to adults (older than 18 years of age) and clinical trials or randomized controlled trials. Abstracts in English only were specified. 

### 1.2. Inclusion Criteria

Studies were selected for inclusion if they had investigated the use of intraoperative local anesthetic infiltration for postoperative pain management following total hip arthroplasty (THA). 

### 1.3. Exclusion Criteria

Studies that did not record either pain scores or analgesic consumption were excluded. Studies that used solely an intra-articular injection (as opposed to a tissue infiltration technique) or a postoperative infusion alone (not preceded by tissue infiltration) were excluded. 

### 1.4. Data Extraction and Analysis

Each study's methodology and results were recorded. Randomized controlled trials (RCTs) were assessed using the Jadad criteria and scored on the 0–5 scale [1]. The Jadad score is based on a three-point questionnaire that asks the following questions: was the study described as randomized, as double blind, and was there a description of withdrawals and dropouts? A point is awarded for each affirmative answer. Additional points are given one, if the method of randomization was described in the paper, and that method was appropriate, and two, if the method of blinding was described, and it was appropriate. Points are deducted if the method of randomization was described but was inappropriate and/or the method of blinding was described but was inappropriate. By these criteria a maximum score of 5 points is possible. A Jadad score (where applicable) of ≥3 was considered acceptable for inclusion. No studies required exclusion based on this criterion. The outcomes (pain scores at rest and on movement, ambulation, length of hospital stay, adverse events) were recorded as defined by the original studies. 

## 2. Results

**Figure 1:**
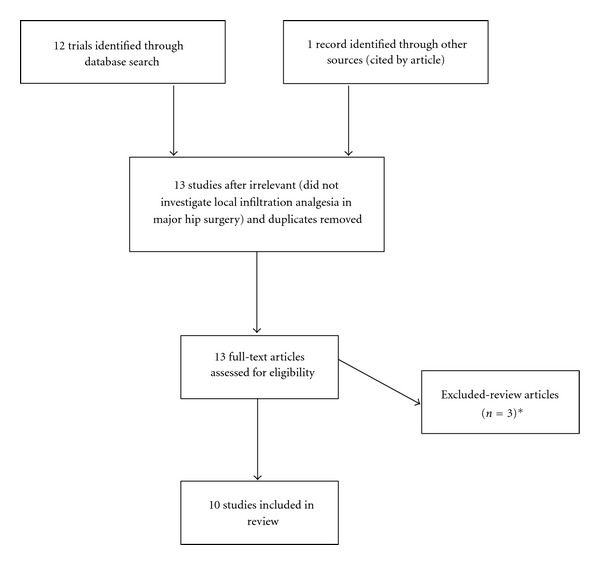
Flow diagram of exclusion of clinical trials for review [40–42].

Ten studies (a total of 893 patients) were identified as pertinent and included for analysis—eight RCTs and two case series. It should be noted that four of these studies looked at a mixt population of patients undergoing hip resurfacing arthroplasty and total knee arthroplasty. The exclusions are outlined in Figure 1. The study characteristics of all articles included are detailed in Table 1.

## 3. Discussion

### 3.1. Introduction

Established methods of postoperative analgesia for total hip arthroplasty are central neuraxial opioids, epidural analgesia, intravenous patient-controlled opioid analgesia (PCA), and peripheral nerve blocks [2]. The prospect collaboration, which aims to provide evidence-based recommendations on a procedure-specific basis, issued guidelines for THA anesthetic management in 2005. The group concluded that there are “various clinical pathways with which to manage pain effectively after THA” and, in summary, recommended use of either general anesthesia plus strong opioids or general anesthesia plus peripheral nerve blockade (femoral or posterior lumber plexus block) or spinal local anesthetic (single shot) plus morphine or epidural local anesthetic plus/minus opioid.

Peripheral nerve blockade (PNB) has been shown to provide equivalent analgesia compared to epidural analgesia with the benefit of a lesser incidence of hypotension and urinary retention [3], however PNB requires a high level of expertise to perform [4]. Recently, in a “pooled data” report of the combined findings of three separate studies of continuous peripheral nerve blockade (CPNB) following total hip (and knee) arthroplasty, a possible causal relationship was suggested between CPNB affecting quadriceps strength and the risk of falling [5]. Choi et al. reviewed the use of epidural analgesia for pain relief following hip (and knee) replacement in a Cochrane review in 2003 and concluded that the beneficial effect of epidural analgesia was limited to 4–6 hours postoperatively, and side effects (hypotension, pruritus, urinary retention) were more frequent with epidural analgesia compared to systemic analgesia [3]. 

With a move towards earlier ambulation of patients following joint replacement surgery, comprehensive analgesia regimes that provide excellent pain relief both at rest and on movement, without reduction in motor function/power and with minimal side effects, are desirable. A number of investigators have found LIA to be superior to placebo/no infiltration and epidural analgesia in terms of postoperative analgesia scores, joint function/rehabilitation, and length of hospital stay. This may explain the increased number of trials investigating the LIA technique in recent years.

### 3.2. Review of the Evidence-Published Trials

Kerr and Kohan published a case series of 325 patients who were given intra- and periarticular infiltration for postoperative analgesia following total hip or knee arthroplasty [6]. The injectate mixture consisted of ropivacaine 2 mg/mL, ketorolac 30 mg, epinephrine 10 mcg/mL. The volume used for THA and hip resurfacing arthroplasty (HRA) was 150–200 mL. The maximum dose of ropivacaine was limited to 300 mg (or 250 mg in some cases). If volumes over 150 mL were required, the injectate was diluted with saline. The mixture was injected into all the tissues of the surgical field in a systematic fashion during surgery. They termed this local infiltration analgesia. The patients were subsequently given a bolus of 50 mL of the mixture (100 mg ropivacaine) at 15 to 20 hours postoperatively via an intra-articular catheter that was sited during the surgery. The authors reported that pain scores were generally satisfactory (0–3/10) and that two thirds of patients did not require morphine during the postoperative period. Most patients were able to walk with assistance between 5 and 6 hours postoperatively. The exact infiltration method as described by Kerr and Kohan was used in a subsequent series of 24 patients undergoing elective HRA and TKA by Otte et al. with similar postoperative analgesic efficacy [7]. 

Parvataneni et al. investigated the use of local anesthetic infiltration as part of a multimodal pain protocol following THA (and TKA) [8]. A mixture containing bupivacaine 40–80 mg and epinephrine (plus morphine, methylprednisolone, and cefuroxime) in a volume of 75–115 mL was used for infiltration. The control patients received intravenous PCA morphine (plus femoral nerve block for TKA patients). Lower pain scores and a shorter length of stay (3.2 versus 4.2 days,  *P* < 0.05) were reported in the THA patients who received infiltration. 

Two studies [9, 10] have compared LIA with placebo saline. Bianconi used ropivacaine 200 mg (40 mL) for infiltration and followed it with an extra-articular infusion of ropivacaine 10 mg/hr for 35 hours [9]. The control group received an extra-articular saline infusion. The LIA group reported lower pain scores at rest and on movement up to 72 hours postoperatively and had lesser opioid consumption. There was a shorter length of stay in the LIA group (6.34 (0.67) versus 8.79 (1.39) days,  *P* < 0.05). Andersen et al. used ropivacaine 300 mg (150 mL) plus ketorolac 30 mg plus epinephrine 0.5 mg for infiltration and followed by, on the first postoperative morning, an intra-articular bolus of ropivacaine 150 mg (20 mL) plus ketorolac 30 mg plus epinephrine 0.5 mg [10]. The LIA group reported lower pain scores from 4 hours up to two weeks postoperatively and lower opioid consumption. Joint function was better in the LIA group at one week but not thereafter. 

One study was published comparing LIA with no infiltration [11]. Busch et al. infiltrated ropivacaine 400 mg (100 mL) plus ketorolac 30 mg plus epinephrine 0.6 mg plus morphine 5 mg [11]. Further infiltration was not given in the postoperative period. The control group received standard care with no infiltration. The LIA group reported lower pain scores in the PACU on movement and lower opioid consumption in the first 24 hours. 

Andersen et al. [12] investigated the analgesic effect of wound infiltration (intraoperative bolus plus top-up via catheter at 8 hours postoperatively) versus epidural analgesia in patients undergoing THA. The patients who received LIA had reduced opioid consumption and length of hospital stay and improved mobilization. Interestingly the LIA group reported significantly lower VAS for pain at both rest and movement from 20 to 96 hours postoperatively, after active pain treatment had ended (20 hours postoperatively).

Specht et al. [13] compared an LIA regimen of intra-operative ropivacaine, epinephrine, and ketorolac infiltration followed by an intra-articular bolus at 10 and 22 hours postoperatively versus a regimen of intra-operative LIA as above followed by a postoperative intra-articular saline bolus in 60 patients undergoing THA. They found no difference in pain scores or opioid consumption between the two groups and a non-significant trend to shorter hospital stay in the intervention group. It should be pointed out that in this study both groups received intra-operative LIA, the difference being in the postoperative bolus drug (LIA mixture or saline). The conclusion from this study was that a postoperative bolus did not seem to offer an additional benefit to intra-operative LIA.

The studies outlined above all found that the LIA technique was an effective analgesic method following THA (superior to placebo/no infiltration or epidural analgesia) and that opioid consumption could be reduced with its use. Three of the studies reported that hospital length of stay could be shortened with the use of the LIA technique [8, 9, 12] and three found a difference in the postoperative functional assessment of the operative hip in favor of the LIA technique [8, 10, 12]. 

Whilst recommended by a number of studies, two recent trials do not advocate the use of the LIA technique in addition to a multimodal analgesic regimen. In a study published in 2011, Andersen et al. investigated the analgesic efficacy of the LIA technique by comparing its use versus placebo in 12 patients undergoing bilateral THA [14]. In this study all patients received intra-operative infiltration of a ropivacaine-epinephrine solution to one hip and 0.9% saline to the other. Supplementary boluses of the solutions used were administered at 8 and 24 hours postoperatively. All patients had a multimodal analgesic regimen (gabapentin, celecoxib, and acetaminophen) commenced preoperatively. The authors reported that postoperative pain scores were low and similar between the hip given ropivacaine and that given saline. They concluded that they could not therefore recommend the LIA technique in addition to the multi-modal approach.

Also published in 2011, Lunn et al. compared the use of LIA (ropivacaine with epinephrine) versus placebo infiltration with saline in 120 patients undergoing unilateral THA, again in the setting of using a preoperatively instituted multimodal analgesic regime of gabapentin, celecoxib, and acetaminophen [15]. The postoperative pain scores in both groups were low (20 (14–38) versus 22 (10–40), ropivacaine versus placebo) and there was no significant difference between the groups (*P* = 0.71). Here again, the authors concluded that they could not recommend LIA as being superior to a multimodal approach. These two studies used only local anesthetic with epinephrine for the LIA. Therefore the possibly confounding effect of ketorolac or another NSAID in the infiltration mixture being responsible for the analgesic benefit with LIA was removed. In both of these studies the authors asserted that the LIA technique may not have a clinically relevant effect when combined with a multimodal analgesic approach and therefore was not recommended. The difference in outcome between the aforementioned trials advocating LIA and these two appears to be related to the use of a comprehensive multimodal analgesic regimen which seems to be as effective as the LIA technique. The LIA technique has been reported to be easy to perform effectively and appears to be safe. Whether or not it provides the most effective analgesia following THA has been questioned however it may have a role in certain subsets of patients such as those who are intolerant of or unsuitable for the multimodal regimen referred to above. Patients who have chronic pain conditions or are habitual opioid users may benefit from the administration of LIA; however these patients are generally not included in studies of postoperative analgesia, and therefore data is lacking. 

### 3.3. LIA Regimens

The systematic infiltration of all tissues in the wound in a staged fashion as the surgery progresses as described by Kerr and Kohan has been widely adopted as the preferred method of infiltration analgesia. The local anesthetic used most often in published work so far is ropivacaine, likely chosen for its reduced cardiotoxicity in comparison to bupivacaine as well as for its intrinsic vasoconstrictor properties [16, 17]. The optimal total dose for maximal analgesic effect and safety has not been determined. Practitioners have reported using doses of 200 mg [9, 12], 300 mg [6, 7, 10], and 400 mg [11] of ropivacaine for infiltration. The use of a fixed recipe or dose is common; the introduction of a dose based on patient weight may be a future consideration.

The postoperative management also has a number of permutations. Most investigators site a catheter for postoperative local anesthetic administration but whether it is better to administer a postoperative bolus dose at a fixed time or a continuous postoperative infusion of local anesthetic is unclear. Bolus intra-articular doses [6, 7, 10, 15] and continuous extra-articular [9] infusions have been used. The timing of administration of the bolus dose, if used, varies. One group gave the bolus at 8 hours [12] while the others waited until the first postoperative day [6, 7, 10]. However, in a study carried out to investigate the effect of adding a local anesthetic infusion via an intra-articular catheter to an intra-operative LIA regime, the authors found no evidence of an improvement in pain scores in the group who received the ropivacaine/ketorolac/epinephrine infusion versus the placebo group and concluded that the catheter delivered infusion could not be recommended [13].

While the placement of a catheter for further administration of local anesthetic is favored, there is no definitive evidence regarding the optimal catheter placement site. Both intra-articular and extra-articular catheters have been used.

The use of adjuvants in the infiltration mixture varies. Nonsteroidal anti-inflammatory drugs (NSAIDs)—mostly ketorolac 30 mg [6, 10–12] which is a directly acting injectable formulation and morphine [8]—have been used, in addition to epinephrine. Steroid (methylprednisolone) has also been used in the infiltration mixture [8]. The contribution these agents make to the analgesic effect of LIA is difficult to determine. Little data exists directly comparing local administration of NSAIDs and opioids with enteral/parenteral administration, and no trial has compared LIA with adjuvants versus LIA without adjuvants. 

The use of a multimodal analgesic approach is an important factor in the successful early mobilization and rehabilitation of patients following THA. It is likely that the success seen with tailored perioperative programmes in shortening time to mobilization and hospital length of stay is due to the comprehensive approach taken to achieve these aims [8, 14, 15]. 

In studies of patients undergoing THA where a comprehensive multimodal analgesic regimen was not used, LIA has been shown to be superior to placebo both in terms of postoperative pain scores and postoperative functional ability [10]. It has not, however, been conclusively proven to be superior to epidural analgesia. In addition, it does not appear to be of value when used in addition to a perioperative multimodal analgesia regime of gabapentin, celecoxib, and acetaminophen. The superior analgesia provided by LIA when compared to placebo has led to secondary benefits, namely, reduction in opioid consumption [9–12], and earlier hospital discharge [8, 9, 12]. Although a number of investigators have examined the use of the LIA technique, most of the studies are small and have not been powered to detect complications such as infection or local anesthetic toxicity. 

### 3.4. Pharmacology of Analgesic Agents Used for Local Infiltration Analgesia

The analgesic effect derived from the local anesthetic can be ascribed to the direct actions of the constituent drug (blockage of ion-gated Na channels on A-delta and C-type nerves and therefore nociceptive nerve endings). However in a number of studies the beneficial effect (both in terms of pain scores and mobility) of the local infiltration analgesia seems to extend well beyond the expected duration of effect of the local anesthetic itself. The anti-inflammatory effect that local anesthetic drugs have been shown to exert may be a factor [18–20]. Local anesthetics reduce the release of inflammatory mediators from neutrophils, reduce neutrophil adhesion to the endothelium, reduce formation of free oxygen radicals, and decrease edema formation [21, 22]. However, a recent study designed to examine the effects of the instillation of the local anesthetic bupivacaine to the surgical wound following Caesarean section found rather unexpectedly that the instillation markedly decreased IL-10 and increased substance P in wound exudates [23]. This may indicate an overall proinflammatory wound response caused by bupivacaine. It would appear that further research in the area is necessary to fully elucidate the effects of local anesthetics agents on the local and systemic inflammatory response. 

### 3.5. Epinephrine

Epinephrine is added for its vasoconstrictor properties which may decrease the systemic absorption of the local anesthetic and also aid in reducing perioperative blood loss. The administration of epinephrine by this technique will result in systemic absorption with resultant alpha- and beta-agonist effects. No deleterious effects have been reported in the literature on LIA in relation to this. The potential for elderly patients or patients with ischemic heart disease to tolerate these systemic effects poorly should, however, not be underestimated.

### 3.6. Nonsteroidal Anti-Inflammatory Drugs

Nonsteroidal anti-inflammatory drugs (NSAIDs) reduce prostaglandin formation. Prostaglandins sensitize nociceptive fibers and lead to amplification and sustaining of pain. However, there is no rigorous data to support a direct peripheral anti-inflammatory effect of NSAIDs. The use of these drugs in the LIA solution may actually be conferring a systemic rather than a local effect.

### 3.7. Opioids

It has been shown that opioids can produce potent analgesia by activating opioid receptors on peripheral sensory neurons [24]. Inflammation causes a number of cellular processes that result in a higher density of opioid receptors at peripheral nerve terminals [25, 26], and this as well as other alterations in intra- and extracellular mechanisms leads to the increased antinociceptive efficacy of peripherally administered opioids in inflamed tissue [27]. 

### 3.8. Pharmacokinetics and Safety Profile

Total hip arthroplasty creates a large incision area with exposed tissue, blood vessels, and bone into which a large volume of local anesthetic is injected with the LIA technique. Such high-dose administration of local anesthetic raises the potential concern of the occurrence of local anesthetic systemic toxicity, a potentially lethal complication.

Only a small number of studies have undertaken blood sampling for serum local anesthetic levels at various time points after the intraoperative infiltration and during subsequent intra- and periarticular joint infusions in an attempt to clarify the pharmacokinetics of local anesthetics administered in this way [9, 11, 28]. Highest total plasma concentrations of ropivacaine seen with the infiltration of ropivacaine 200 mg followed by an infusion at 10 mg/hr were between 0.30 and 1.28 mcg/mL, with a mean of 0.71 mcg/mL [9]. Stringer and colleagues carried out a study to investigate the pharmacokinetic and safety profile of ropivacaine 360–400 mg given via wound infiltration followed by a continuous wound infusion of 300 mg over 48 hours (6.25 mg/hr) started 12 hours postoperatively in patients undergoing THA (and TKA) [28]. The bolus infiltration resulted in peak plasma levels below 2 mcg/mL until the infusion was commenced which increased levels to up to 4.36 mcg/mL (range of peak 0.65 to 4.36 mcg/mL). Although the peak levels during the infusion were in excess of previously reported safe thresholds, there was no clinical evidence of toxicity. 

 The question of safe plasma levels of the various local anesthetic agents arises. One study on volunteers reported the toxic level of ropivacaine to be 0.6 mcg/mL (range 0.3–0.9 mcg/mL) [29]. Other investigators have found no systemic toxic effects with peak plasma levels, in patients receiving ropivacaine infusion, of up to 3.70, 6.08, and 7.1 mcg/mL [30–32]. The reported toxic level of bupivacaine is 0.3 mcg/mL (range 0.1–0.5 mcg/mL) [29]. 

### 3.9. Complications

Information on the incidence of potential complications is lacking due to the comparatively recent introduction of the technique and the small numbers of patients enrolled in published trials.

### 3.10. Infection

Concerns have been raised about the potential for infection in the presence of an indwelling catheter [33]. In a published collection of case reports, 34 reports describing adverse events in 40 patients during the use of continuous infusion pumps delivering local anesthetic were documented. Eighteen of these patients (45%) had undergone orthopedic surgery. The most commonly reported complication was tissue necrosis (*n* = 17, 42.5%) followed by surgical wound infection (*n* = 15, 37.5%). The addition of epinephrine to the local anesthetic infusion was postulated as a potential cause. The nature of the reports, however, does not allow definitive conclusions regarding a causal link between the presence of the infusion device and the complication. A number of authors using the LIA technique describe using an epinephrine-containing solution for the deep tissue infiltration and a solution without epinephrine for the subcutaneous tissue infiltration.

 In a review of continuous wound catheters for all types of surgery, the overall incidence of infection was reported as 0.7% in active group catheters and 1.2% in the nonactive or control group catheters [34]. Nearly all patients had a catheter placed; therefore comparison with noncatheter groups was not possible in this review. In the study by Bianconi et al. the catheter tips underwent microbiological analysis after removal at 55 hours, and the results were negative for growth in the two groups (ropivacaine and saline infusions) [9]. 

A study of patients receiving postoperative wound infusion following total knee arthroplasty reported one deep wound infection in the series of 154 patients [35]. 

The published work investigating the use of indwelling catheters for administration of local anesthetic so far has not been powered to detect if the catheters pose an added infection risk. It would seem prudent to be cognizant of the fact that they represent a potential source of infection and as such should be inserted under sterile conditions and any boluses given using aseptic technique. How long it is safe to leave a catheter in situ postoperatively remains unclear.

### 3.11. Local Anesthetic Systemic Toxicity

The studies that investigating the LIA technique have not reported any instances of local anesthetic toxicity despite the infiltration of large doses of local anesthetic. However, these are small studies on a limited number of patients, and consequently this cannot be extrapolated to mean that there is no risk of local anesthetic systemic toxicity.

### 3.12. Chondro- and Myotoxicity

Local anesthetic agents can be directly toxic to tissues. Continuous infusion of local anesthetic agents into joint cavities has been linked with subsequent chondrolysis [36–38]. It has been postulated that this direct toxic effect may be related to the modulation of the local inflammatory response by local anesthetic agents [23]. Chondrotoxicity has not been reported following the use of LIA for THA. Patients undergoing this surgery usually have preexisting severe degeneration of the joint with associated loss of cartilage, hence the need for joint replacement. Myotoxicity—in the form of myopathy—has been reported with local anesthetic use [38, 39]. It is a rare but sometimes serious side effect of local anesthetic application. In comparison with ropivacaine, the extent of bupivacaine-induced muscle lesions was significantly larger in one experimental study [38].

### 3.13. Limitations

The limitations of this paper should be noted. The RCTs investigating LIA to date are small single-centre studies. Other studies may have recruited larger numbers, but these were in the form of retrospective series. The RCTs detailed in this paper vary in their design and quality. Only a proportion of these studies were double-blinded therefore the risk of bias in the outcome reporting of the other studies cannot be excluded. This paper only included studies published in English introducing an inherent publication bias.

## 4. Conclusion

In conclusion, the existing data regarding the use of local infiltration analgesia following total hip arthroplasty consists of the results from a relatively small number of small-moderate-sized clinical trials. The LIA technique has been shown to be an effective analgesic method. It has been proven to be superior to no infiltration, placebo saline infiltration and, in one study, epidural analgesia. It has not been shown to provide additional analgesic or outcome benefit in the setting of a comprehensive multimodal analgesic approach but can be regarded as an effective analgesic method following THA, and consideration should be given to its use by the surgeon and the anesthetist in the planning of the analgesic management strategy for this surgical procedure.

## Figures and Tables

**Table 1:** Published trials examining use of local infiltration analgesia following total hip arthroplasty.

Author, year	Type of surgery	Study design	Intervention	Control	Pain scores	Opioid consumption	Function	Length of stay	comment
Andersen et al. [12] 2007	THA	RCT *n* = 80	LIA 100 mL: ropivacaine 200 mg + epi 0.5 mg + ketorolac 30 mg IA bolus 20 mL (ropivacaine 150 mg + epinephrine 0.5 mg + ketorolac 30 mg at 8 hrs)	Epidural to 20 hours	Lower VAS LIA group from 20 to 96 hours *P* < 0.001 to *P* = 0.04 (variable with time)	Reduced in LIA group *P* = 0.04 for first 20 hrs, *P* = 0.05 for total study period up to 96 hrs	More patients able to walk at 8 hours in LIA group *P* < 0.001	Reduced LIA group 4.5 (3–6) versus 7 (5.5–7) days (mean, range), *P* < 0.001	Jadad score = 3 96 hour followup

Andersen et al. [10] 2007	THA	RCT *n* = 40	LIA 150 mL: ropivacaine 300 mg + ketorolac 30 mg + epinephrine 0.5 mg IA bolus next morning 20 mL of mixture	Saline infiltration and injection	Lower in LIA group from 4 hours up to 2 weeks *P* < 0.001 to *P* = 0.04 (variable with time)	Lower in LIA group *P* < 0.001 to *P* = 0.01 (variable with time)	Better at 1 week with LIA (*P* = 0.02), no difference after	No difference	Jadad score = 4 6-week followup

Andersen et al. [14] 2011	Bilateral THA	RCT *n* = 12	LIA: ropivacaine 340 mg + epinephrine 1.7 mg to one hip	Saline to control hip	Low, similar between hips		N/R	4 days median	Jadad score = 4 48-hour followup

Bianconi et al. [9] 2003	TKA and THA Results pertain to THA (*n* = 29)	RCT *n* = 37	LIA 40 mL: ropivacaine 200 mg Extra-articular infusion ropivacaine 10 mg/hr for 55 hrs	Extra-articular saline infusion	Lower scores in LIA group at rest and on movement up to 72 hours *P* < 0.05	Lower in LIA group *P* < 0.05		Reduction in length of hospital stay in LIA group 6.34 (0.67) versus 8.79 (1.39) days (mean, SD) *P* < 0.05	Jadad score = 4 72-hour followup

Busch et al. [11] 2010	THA	RCT *n* = 64	LIA 100 mL: ropivacaine 400 mg + epinephrine 0.6 mg + ketorolac 30 mg + morphine 5 mg	Nil	Lower VAS on movement in PACU in LIA group *P* = 0.0019	Lower PCA use first 24 hours in LIA group *P* = 0.0093			Jadad score = 5 6-week followup

Kerr and Kohan [6] 2008	TKA THA HRA Results pertain to all surgery types not only THA (*n* = 54)	Series *n* = 325	LIA 150 mL: ropivacaine 300 mg + epinephrine 1.5 mg + ketorolac 30 mg IA bolus 50 mL of mixture at 15–20 hours	No control	Pain control satisfactory with NRS of 0–3/10	2/3 of patients did not require opioid analgesia			

Lunn et al. [15] 2011	THA	RCT *n* = 120	LIA: ropivacaine 300 mg + epinephrine 1.5 mg Multimodal analgesic regime	Saline infiltration multimodal analgesic regime	No difference between groups *P* = 0.71	No difference between groups *P* = 0.45	N/R	N/R	Jadad score = 5 8-hour followup

Otte et al. [7] 2008	TKA HR Results pertain to HRA group	Series *n* = 24	LIA150 mL: ropivacaine 300 mg + epinephrine 1.5 mg	No control	VAS scores at rest: HRA ≤ 33				Replication of Kerr and Kohan technique

Parvata-neni et al. [8] 2007	THA and TKA Results pertain to THA group (*n* = 60)	RCT *n* = 131	LIA: bupivacaine 200–400 mg + morphine 4–10 mg + epinephrine 0.3 mg + methylprednisolone 40 mg + cefuroxime 750 mg + saline 22 mL	TKA: FNB + PCA THA: PCA No infiltration	Lower pain scores in THA patients in LIA group *P* < 0.05		More patients able to SLR on POD 1 in LIA group 52% versus 15%, *P* < 0.05	THA patients in LIA group had reduced length of stay 3.2 versus 4.2 days (mean), *P* < 0.05	Jadad score = 3 3-month followup

Specht et al. [13] 2011	THA	RCT *n* = 60	LIA: ropivacaine 200 mg + ketorolac 30 mL + epinephrine 1 mg (102 mL) 51 mL of the solution via IA catheter at 10 and 22 hrs multimodal analgesic regime	Also LIA. Saline via catheter at 10 and 22 hrs multimodal analgesic regime	No difference between groups *P* = 0.04 to *P* = 0.1 Further statistical analysis meant that this result “should be regarded as insignificant” as per authors	Similar between groups *P* = 0.5	N/R	Trend to shorter in intervention group, NS 3 (2–6) versus 3 (2–7) days (mean, range) *P* = 0.09	Jadad score = 5 Followup 3 days

THA: total hip arthroplasty, HRA: hip resurfacing arthroplasty, TKA: total knee arthroplasty, NRS: numerical rating scale, POD: postoperative day, RCT: randomized controlled trial, N/R: not reported, NS: not statistically significant.
